# Impact of an improved outdoor space on people with dementia in a hospital unit

**DOI:** 10.3389/frdem.2024.1404662

**Published:** 2024-06-06

**Authors:** Lorraine Ng, Suzanne M. Dyer, Suzanne Dawson, Kate Laver

**Affiliations:** ^1^The Caring Futures Institute, Flinders University, Bedford Park, SA, Australia; ^2^Mental Health Services, Southern Adelaide Local Health Network, SA Health, Daw Park, SA, Australia; ^3^Flinders Health and Medical Research Institute, Rehabilitation, Aged and Extended Care, College of Medicine and Public Health, Flinders University, Bedford Park, SA, Australia; ^4^Southern Adelaide Local Health Network, SA Health, Daw Park, SA, Australia

**Keywords:** Alzheimer's disease, behaviour, BPSD, dementia, falls, garden, hospital, outdoor

## Abstract

**Introduction:**

Gardens and outdoor spaces are an important part of institutional environments for people with dementia. However, evidence regarding the benefits these spaces have for people with dementia is still limited. This paper presents the evaluation of the redevelopment of an inaccessible outdoor space into a therapeutic garden on a high dependency psychogeriatric unit in an acute hospital.

**Method:**

A Mixed methods evaluation was undertaken. An interrupted time series analysis investigated the impact of the garden on falls and challenging behaviours of patients using routinely collected data. Perspectives of the redeveloped garden were captured through (a) a staff survey and (b) semi-structured interviews with families of patients.

**Results:**

Rates of falls and challenging behaviours dropped at the time of the garden opening but showed increasing rates each month both before and after the garden opened. Most staff believed that the garden provided benefits for patients however limited staff time and concerns over patient safety were barriers to use. Families identified four main themes related to garden use including: (1) being outside (2) occupation and identity, (3) being stimulating, and (4) barriers and facilitators.

**Conclusion:**

The garden was regarded positively by families and staff however, there were barriers that prevented it from being better utilised. Staff concerns over risk were not reflected in falls and challenging behaviour outcomes. Further research into how barriers to garden use may be overcome is justified.

## 1 Introduction

Natural environments have been used to promote health and wellbeing for a large part of the history of modern medicine. Florence Nightingale is widely known for promoting the use of sunlight and fresh air in the 1860's (Sternberg, 2009). Since the 1980's, research has more frequently identified the benefits of natural environments for people with health conditions such as those experiencing post-operative recovery (Ulrich, 1984).

It is well-recognised that the physical environment plays an important role in the wellbeing of people with dementia (Fleming et al., 2020). Environmental adaptations for people with dementia have been found to support performance in activities of daily living in a wide range of areas (Woodbridge et al., 2018). Architectural design that supports people with dementia is also increasingly recognised as important in hospitals and other care settings (Harrison et al., 2022; Golembiewski, 2023). However, there is a need for further research on the role of environmental design to support people living with dementia in acute hospital settings (Røsvik and Rokstad, 2020).

It is important that garden and outdoor spaces in health settings are also well-designed and support the function of people with dementia (Ng et al., 2023). Guidance on the design of garden and outdoor spaces has emerged over the last decade which has been of use to designers, health and aged care organisations and clinicians (Pollock and Cuningham, 2012). Important features of garden design for people with dementia are those that promote orientation, accessibility, meaningful activity, reminiscence, sensory stimulation and safety (Cochrane, 2010). However, despite ongoing research, evidence regarding the benefits received by people with dementia from using outdoor spaces is limited (Ng et al., 2023).

This project took place on the high dependency psychiatric unit for older persons at the Queen Elizabeth Hospital in South Australia. The unit supports people with behavioural and psychological symptoms of dementia as well as people 65 and over who have acute mental health conditions and need high intensity care. Due to the diagnoses and comorbidities experienced by patients using the service, the unit had a high rate of falls and challenging behaviours. The physical environment was generally considered pleasant by visitors but had limited indoor or outdoor environmental features to support physical reconditioning, occupational participation or sensory experiences. The unit had one large existing garden which incorporated concrete, artificial grass, ligature safe benches and a garden bed. In the year 2020, the site received funding to re-develop an unused and inaccessible outside area into an additional garden space for the unit. The intention of the garden was to (1) enable patients to spend time in a therapeutic outdoor environment, and (2) provide opportunities for mobility training, maintenance, and rehabilitation.

The design and development of the garden occurred over 16 months and was conducted by the unit's multidisciplinary team, led by the occupational therapist with close nursing and physiotherapy involvement. A consultative process was used, involving appraising other therapy gardens, discussion with local experts and feedback from patients and their families to inform design of the garden. In addition, a scoping study was carried out by Ng et al. (2023) to identify current literature that supported outdoor activity engagement. The garden design goal was to provide a garden environment which assisted patients to achieve therapeutic goals around self-regulation, occupational engagement and the maintenance or rehabilitation of strength and mobility to facilitate recovery and discharge.

**Figure 1:**
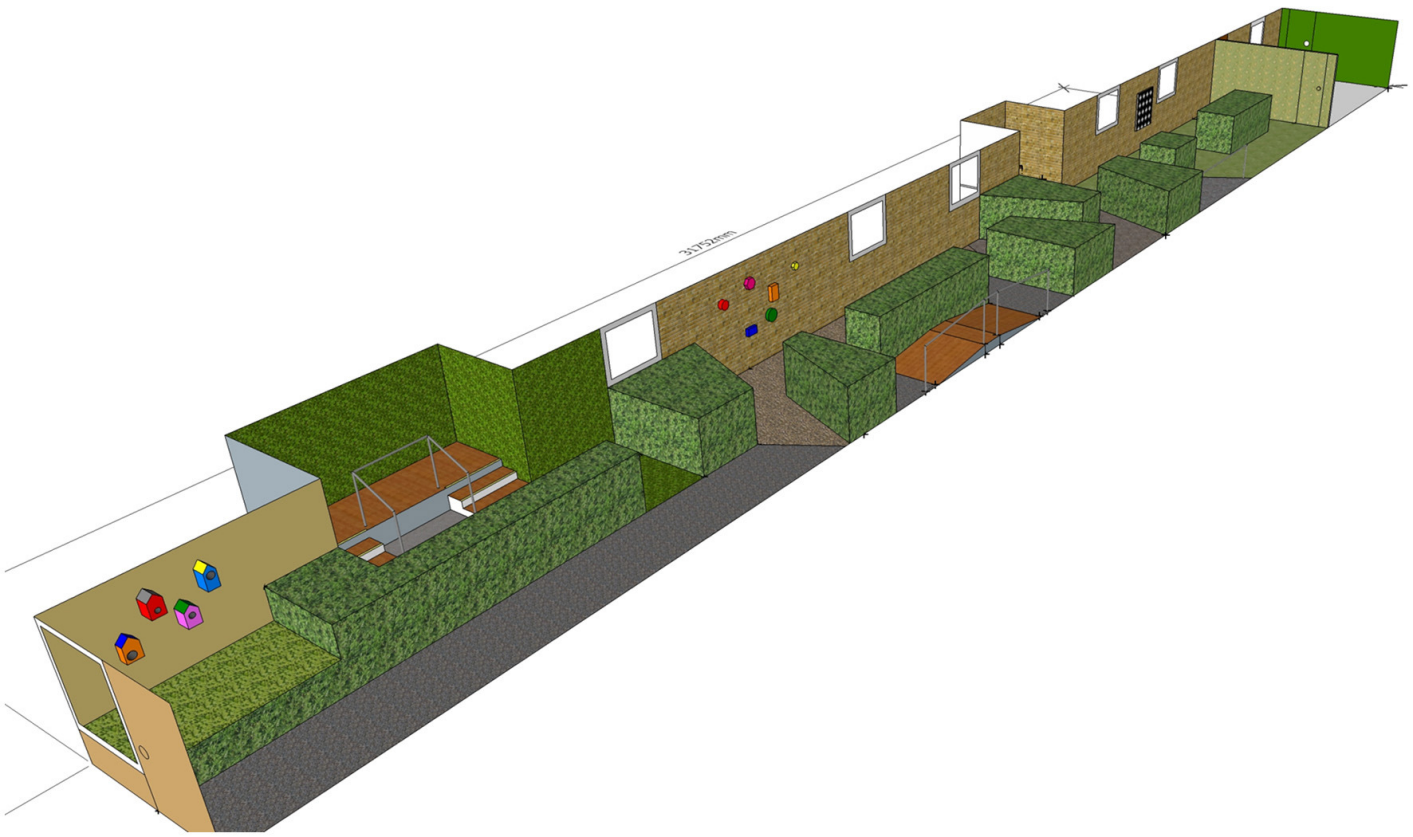
Planned design for the garden redevelopment.

The new garden space incorporated sensory modulation concepts and mobility garden design ideas to promote increased physical strength and balance. These included non-linear walking paths, signposting features to draw patients through the garden, varied and loose path surfaces to provide sensory input and challenge balance, mobility obstacles including a bridge and stairs, interactive features mounted on walls and trees, sensory plantings, seating, and raised garden beds. See Figure 1 for the garden design.

**Figure 2:**
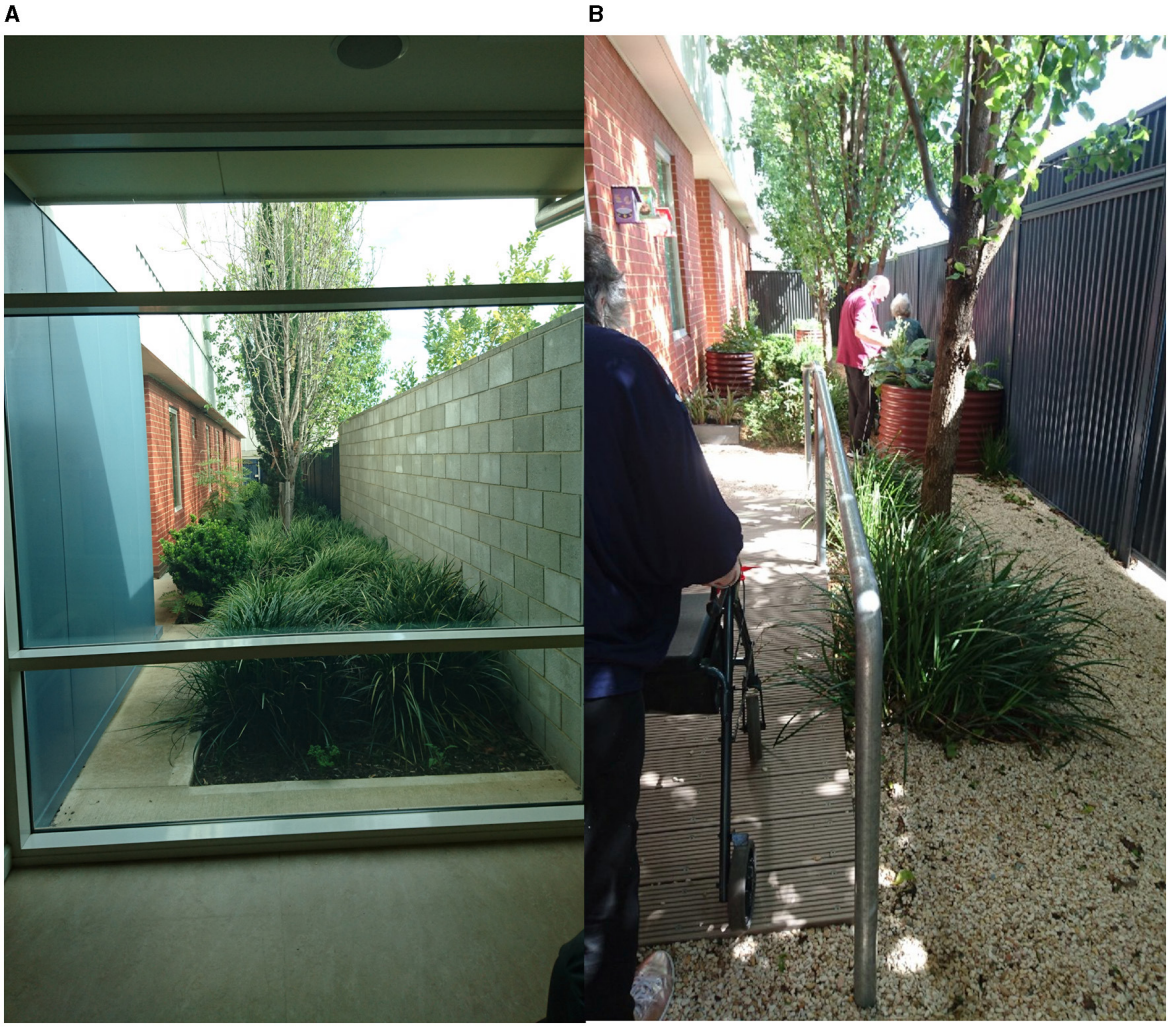
The outdoor space **(A)** before redevelopment; **(B)** in use after redevelopment.

The new garden space was opened for use by patients and staff in December 2020. Due to the requirements for ligature risk management, patients required supervision while spending time in the garden. Staff were encouraged to use the garden as part of usual care. Patients also used the garden during clinical assessments as well as for therapeutic interventions including sensory modulation, distress management, mobility and strength training, behavioural activation, or functional retraining. See Figure 2 for images of the redeveloped outdoor space.

This study describes an opportunistic mixed methods evaluation of the redeveloped garden with the aim to explore the impact of the re-design of this space. The objectives of the study were to understand:

- Does access to a garden designed to support mobility and activity engagement on a high dependency unit result in fewer falls or challenging behaviour incidents while patients are on the unit?
- What are the perspectives of staff regarding the garden space?
- What are the perspectives of families regarding the garden space?

## 2 Methods

### 2.1 Design

This mixed methods study comprised three components: an interrupted time series analysis, interviews with families and a survey of clinical staff based on the unit.

Ethics approval was granted by the Central Adelaide Local Health Network (CALHN) Human Research Ethics Committee (Ref no.: 13714).

### 2.2 Interrupted time series

Interrupted time series analyses allow causal effects to be identified in situations where it is not possible to have a prospective control population (Penfold and Zhang, 2013). Data recorded on the unit pre and post redevelopment of the garden was compared to identify if trends that occurred following the intervention show a difference to those that might have occurred if the intervention had not been implemented (Kontopantelis et al., 2015). Aggregate data was collected from the hospital's Safety Learning System where all falls and challenging behaviour incidents for the unit are routinely reported. Falls reports occur when a patient unintentionally comes to rest on a lower surface or where this would have occurred without outside intervention. Challenging behaviour incidents were defined as where a person threatened or attempted to do harm to themselves, others or property. They did not include situations where security staff were pre-emptively called to enforce care against a persons will. Monthly data on the number of falls and behavioural incidents in all areas of the unit were collected for six time points before the introduction of the garden and six following its introduction for the entire unit. As the outdoor space was not accessible to patients prior to redevelopment, data on the falls and behavioural incidents occurring within the garden were available only for the 6 months following the redeveloped garden opening. Due to the variability of the number of beds that may be occupied in the unit and the small numbers of patients on the unit, the data were analysed as number of falls or challenging behaviour incidents per 100 occupied bed days. Data were analysed using ordinary least-squares regression with Newey-West standard errors (lag1) using the statistical programme STATA (version 18.0) which adjusts for autocorrelation and is suitable for the short series of data available (Turner et al., 2021).

### 2.3 Staff surveys

Staff (allied health, medical, and nursing) who were employed by the health service and had worked on the unit since the opening of the garden were invited to participate in an online survey distributed via the email distribution list. The survey was developed for this study and made available via Qualtrics software. It is provided as Supplementary material. Questions were based on outcomes reported in other studies of outdoor spaces for people with dementia. The short survey took ~5 min to complete and the 17 questions included single selection questions, multiple selection questions and free text questions. Data were collected in five subsections: (1) participants demographic information; (2) observations of garden usage; (3) factors that influence garden use; (4) participants opinions; (5) perceived risks. The survey was made available online and in paper copy with participants advised that implied consent was given through participation and that results would remain anonymous. For paper copies staff members were asked to place the completed survey directly in the occupational therapist's pigeonhole with no identifying information to maintain anonymity.

Data were extracted from Qualtrics for each question and were imported into an Excel spreadsheet. Descriptive statistics were used to summarise data and themes were identified by categorising responses to the free text questions.

### 2.4 Family interviews

Interviews were used to explore the perspectives, opinions and experiences of families on behalf of patients with dementia, as the majority of patients did not have the cognitive or language capacity to participate in an interview. A qualitative descriptive approach was used to analyse the data (Stanley, 2015). Project staff approached eligible family members who were the primary contact person for a patient, over 18, able to give informed consent, spoke sufficient English to participate in the interview and had observed their family member spend time in the garden. Family members were offered the opportunity to participate in an in-person semi structured interview with the ward occupational therapist (LN) at their preferred location, during or after their family member's admission, depending on their choice. Research questions were based on an interview guide developed by the researchers and informed by the findings of other studies on use of outdoor spaces by people with dementia.

Interviews were audio recorded and professionally transcribed verbatim. Data and transcripts were thematically analysed using Braun and Clarke's (2006) process of reflexive thematic analysis. Two researchers (KL and LN) independently familiarised themselves with the data and conducted early coding. The researchers then discussed initial codes before continuing to independently code the data set using agreed codes. Themes were then generated through inductive analysis to answer the research question regarding perspectives of families on the garden space. Data were managed using a combination of manual coding and NVivo (QSR International, release 1.3) electronic coding management software.

## 3 Results

### 3.1 Falls

During the 6 months prior to the new garden opening, 10 patients experienced 30 falls on the unit, with the most falls by a single person being 14. After the opening of the garden, 32 falls were experienced by five patients with the most by a single person being 20 falls. No falls occurred in the new garden space during the 6 months of data collection after it was made accessible to patients.

**Figure 3:**
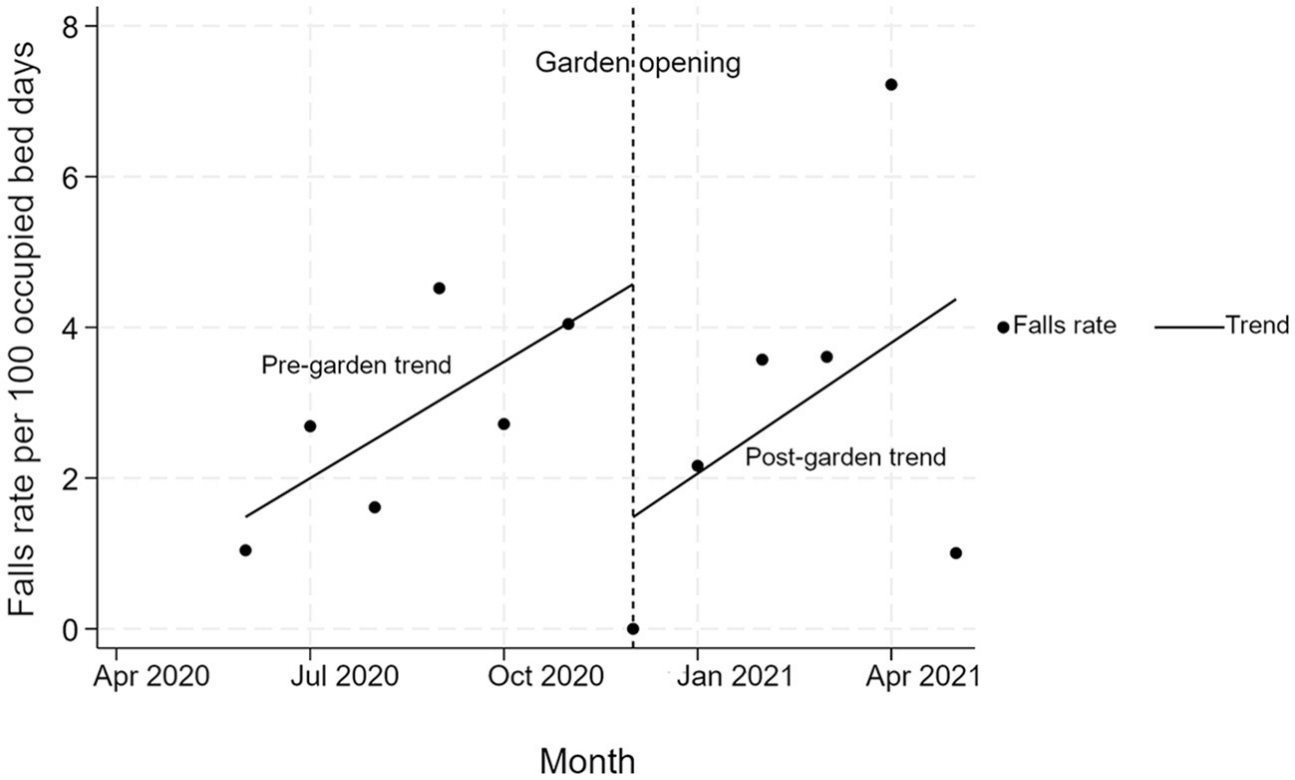
The rate of in falls per 100 bed days per month prior and following the opening of the new garden.

A monthly increase of 0.5 falls per 100 bed days [95% confidence interval (CI): 0.3–0.8; *p* = 0.001] was observed prior to the opening of the garden. A step decrease of 3.1 falls per 100 bed days was seen at the time of the garden opening (95% CI: −6.1 to −0.1; *p* = 0.044). After the garden opened there was a non-significant increase in the monthly rate of falls per 100 bed days by 0.1 (95% CI: −1.3 to 1.4; *p* = 0.917), reflecting an overall increase in falls of 0.6 a month per 100 bed days (95% CI: −0.8 to 1.9; *p* > 0.347) over the 6 months after the garden opened. See Figure 3.

### 3.2 Challenging behaviours

A total of 27 challenging behaviours experienced by 12 patients were reported in the 6 months prior to the opening of the garden, with six challenging behaviours being the maximum number by one person. In the 6 months following the opening of the garden, six patients experienced 16 challenging behaviours, with one individual experiencing five challenging behaviours. No challenging behaviours occurred in the new garden space during the 6 months of data collection after it was made accessible to patients.

**Figure 4:**
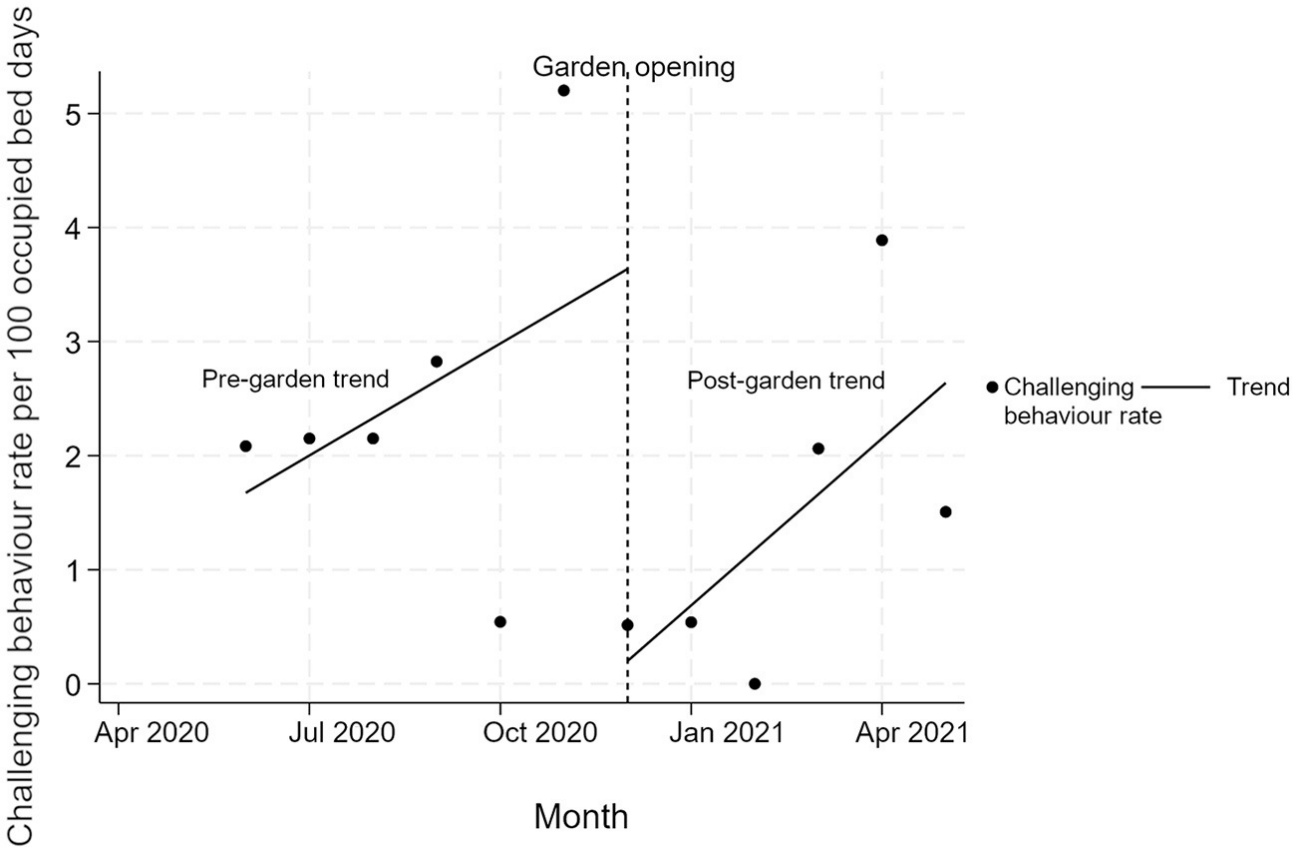
Interrupted time series analysis of change in challenging behaviours per 100 bed days over 12 months.

Challenging behaviours increased by 0.3 per 100 bed days each month (95% CI: −0.4 to 1.0; *p* = 0.32) prior to the opening of the garden. In the 1st month of the garden opening there was a significant drop of 3.4 challenging behaviours per 100 bed days (95% CI: −6.7 to 0.2; *p* = 0.042). However, this was followed by a non-significant increase of 0.1 (95% CI: −0.8 to 1.1; *p* = 0.69) in the rate that challenging behaviours per 100 bed days increased each month compared to the time before the garden opened. This led to a monthly increase of 0.4 challenging behaviours per 100 bed days per month (95% CI: 0.0–1.0; *p* = 0.049) over the 6 months after the garden opened. See Figure 4.

### 3.3 Staff survey

Twelve staff (out of an eligible 36 staff) participated in the survey, including five medical or psychiatric professionals, five nurses and two allied health staff. Almost half (42%) had worked on the unit for < 12 months and 33% had worked on the unit for between 1 and 3 years. Only one staff member had regularly spent time in the garden with patients while four occasionally spent time with patients in the garden and seven reported they had never done so. Of the people observed using the garden, 92% reported seeing patients and allied health staff, 33% reported observing nurses, 25% observed families, and 17% observed doctors and patient's friends, respectively.

**Figure 5:**
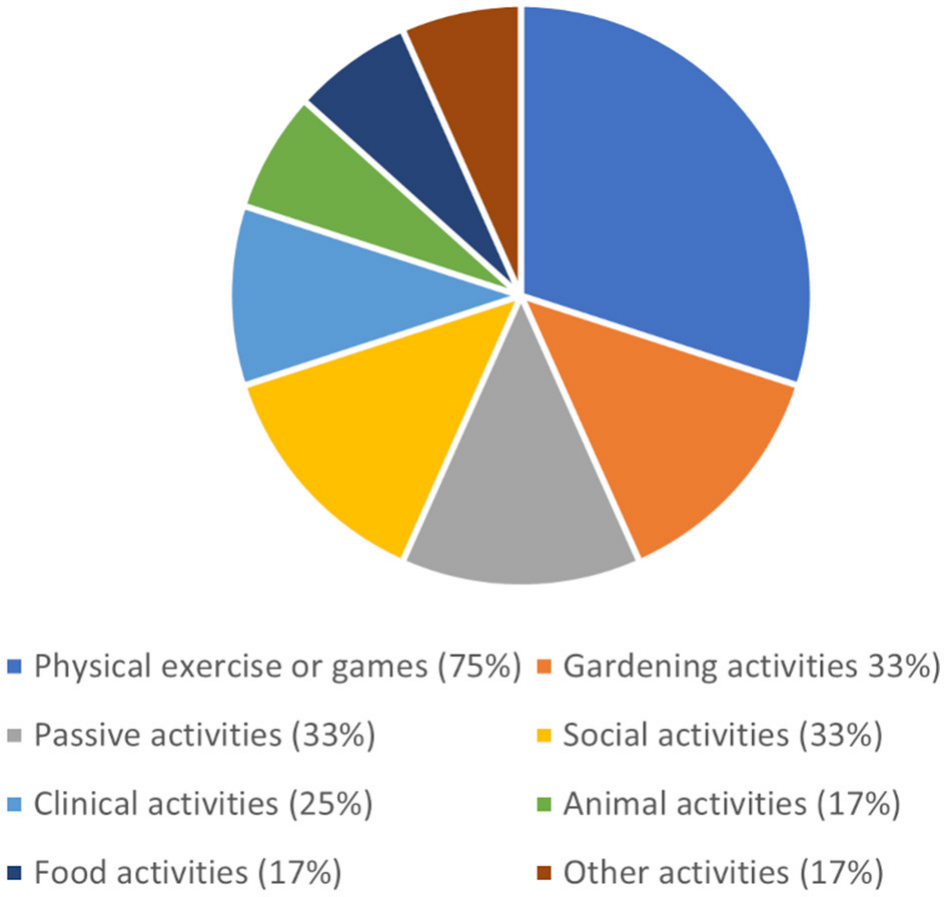
Percentage of staff observing different types of patient activities in the garden.

The most common type of occupation that staff observed patients participating in was physical exercise which was observed by 75% of respondents. Garden related activities, passive activities and social activities (33%) were also reported while some staff reported observing patients participating in clinical activities such as assessments (see Figure 5). The most common specific occupations staff observed patients participating in were practising walking (67%), looking at the garden scenery (42%), talking to others (33%), practising steps (33%), and exercising (33%).

Responses indicated that the garden was thought to improve the unit in a variety of ways with all but one staff member reporting it added sensory experiences (92%). More than half of the staff felt it added activity options (75%), connexion with nature (75%), fresh air (75%), sunlight (67%), assessment options for staff (67%), intervention options for staff (58%), and an attractive view (58%). Five staff members felt that it provided cognitive support and four staff felt it attracted birds and insects as a positive addition to the unit. Factors that prompted staff to consider taking a patient into the garden was closely split between patient interest, mood based and clinical factors with the most common being expressing interest through looking at the garden (15%) or asking to go out (13%) and the least likely being if the patient was angry (3%). Staff expressed their preference to use the garden over alternate outdoor spaces to offer a stimulating environment or as an alternate environment to remove them from stressful situations.

“*If I had more time/during a code blue situation I often provide assistance to consumers distressed by the code—I would consider taking them to the garden in the future as it would provide a good distraction” (respondent 8)*.

Staff also described how the garden added to a positive work environment by providing additional options to patients, families and staff.

“*It offers an alternative environment to offer consumers, provide a private area for 1:1 assessment or families to walk through with consumers, offers opportunity for consumers to reminisce on flowers/garden of their own” (respondent 3)*.

“*It adds to the therapeutic modalities we have at our disposal. It promotes non-biological therapy” (respondent 5)*.

However, one respondent did not feel it added benefit to the unit.

“*It's not safe for the consumers to walk and the people have deconditioning health issues including mobility and mental state” (respondent 2)*.

**Figure 6:**
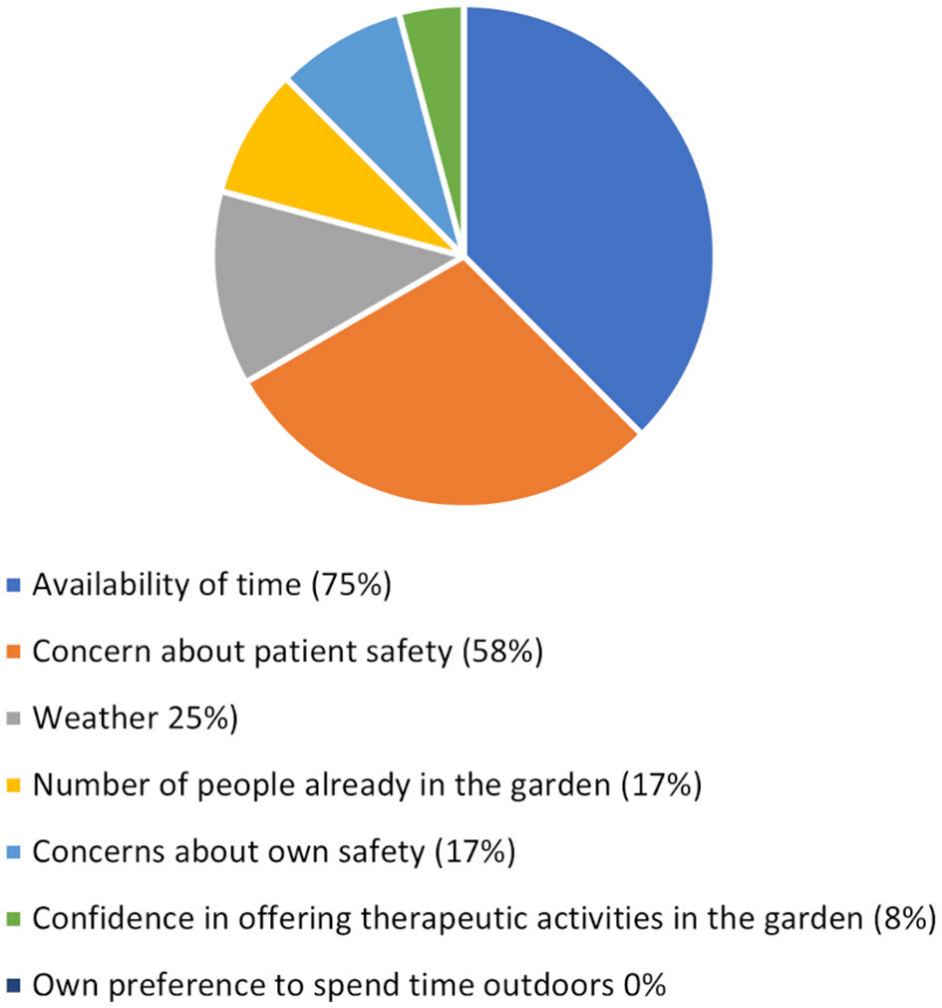
Percentage of staff reporting factors that influenced their use of the garden.

Staff reported that the factor that most heavily influenced their ability to use the garden with patients was the availability of their time (75%) followed by concerns of patient safety (58%; see Figure 6). Safety risks that staff identified they would be concerned about included falls (75%), aggression (33%), and challenging behaviours (33%) with single respondents identifying concern over the cold temperature, cuts and abrasions, injuries and sunburn. Overall, the majority of respondents rated the safety of the garden as safe (33%) or very safe (33%), with 17% rating it risky and a single respondent (8%) rating it very risky.

As described in Table 1, staff's opinions of the garden tended to be positive (75–84%). However, a small number of respondents somewhat or strongly disagreed with this (8–16%).

**Table 1:** Impacts on patients and opinions on the garden.

	**Strongly agree**	**Somewhat agree**	**Neither agree or disagree**	**Somewhat disagree**	**Strongly disagree**
Garden has a positive impact on patients' mood/behaviour while they are using it	7 (58%)	2 (17%)	1 (8%)	1 (8%)	1 (8%)
Garden has a positive impact on patients' mood/behaviour after they have used it	5 (42%)	5 (42%)	1 (8%)	0	1 (8%)
Most patients like the garden	5 (42%)	2 (17%)	3 (25%)	1 (8%)	1 (8%)
Most families and visitors like the garden	5 (42%)	4 (33%)	1 (8%)	1 (8%)	1 (8%)
Most staff on our unit have a positive opinion about using the garden	4 (33%)	3 (25%)	3 (25%)	1 (8%)	1 (8%)
Would recommend a similar garden to staff from another unit	5 (42%)	4 (33%)	1 (8%)	1 (8%)	1 (8%)
Spending time in the garden is likely to be beneficial for the persons mobility/physical function	7 (58%)	2 (17%)	2 (17%)	0	1 (8%)

Suggestions regarding improving the garden included paving the area to reduce falls risk and incorporating the garden into therapeutic plans and assessments.

### 3.4 Family interviews

Interview participants included six family members with four being spouses, one a child and one a sibling. Ages ranged between 51 and 76 years and three participants were female and three participants were male. One patient was able to participate with her spouse and one patient was present but not able to participate. All family members were supporting patients with a diagnosis of dementia who experienced behavioural and psychological symptoms. All family members who agreed to be contacted by the researcher consented to participate with one withdrawing prior to the interview due to competing time demands. Interviews lasted between 20 min and 1 h. Analysis of the interview data identified four main themes.

#### 3.4.1 Being outside

The garden was considered to be beneficial and a positive experience for patients by all participants. This concept encompassed being outside as a positive in itself and also the feeling and opportunities that came with it. Participants also contrasted being in the garden to spending time in other spaces on the unit.

“*It's probably just being out there and again in the sun and garden and makes you happier… Than being inside in a building… Especially a hospital, not being rude, but it's… fairly boring. So it's nice to get out and see some different things and different surroundings” (participant 3)*.

When discussing between themselves what it was like being in the garden participant 1 and his spouse who was a patient shared “*You felt like you were free. You weren't walking around like a zombie inside the ward itself (participant 1).”*

#### 3.4.2 Occupation and identity

Time in the garden was seen to link patients to their identities outside of the hospital space and to allow them to express this in positive ways that were not possible elsewhere on the unit. It gave them opportunities to engage in occupations that confirmed and reinforced their self-identity through participation and achievement. When one participant was asked what he felt his wife liked about the garden he responded:

“*I think she just enjoyed doing things… Yeah. Well, nothing else to do around there in the ward itself… I think getting out there and picking up leaves and sweeping and everything else was something because she could... I think the main problem she feels like she's a bit... She probably feels a bit useless. She feels like not contributing to anything” (participant 5)*.

All participants commented on how the garden had facilitated patients to connect to their identity. The garden environment allowed activity engagement that gave patients a sense of purpose and meaning.

“*Well, he's doing what he enjoys doing and he's feeling useful. Being able to sweep the leaves up and just, yeah. Basically being... Felt like he was able to help somebody” (participant 2)*.

“*Because at home with the dementia, the garden was his. And he would sweep every leaf from that garden even off the dirt, the lawn, everywhere. And I'd let him go, he'd chop things, he'd put things …. around and stuff like that. But that was his... and he spent nearly every day out there… That was important for him to do that, very” (participant 6)*.

“*He's not a real outdoor person, but I think, I'm pretty sure that he may be felt he was achieving something perhaps… Making an achievement by going out there and being able to move on those different surfaces. And I think he felt happy with the results as well” (participant 3)*.

#### 3.4.3 Stimulating

All participants described the garden as an environment that supported patient wellbeing through providing beneficial stimulation by allowing patients to have experiences that were not possible in other areas of the unit.

Physical stimulation was considered important by most participants. Some recognised the value of the garden features to assist in working towards physical goals while for others the stimulation of exposure to different physical experiences was considered beneficial in itself.

“*Yeah. Its stimulating… There's no, probably, other word for it… And with the occupational therapy, going up and down and stairs and all that sort of stuff. Yeah. You need to (participant 1). And it's preparing for when you come home, I think (patient).”*

“*The time we went out there and he got to one end of the other and seemed to be walking on all the different textures of ground really well, up and down the steps, the ramps, and everything? Yeah, I thought it could be really effective” (participant 4)*.

“*So he actually had to do some work… Which obviously is good, but he was always a bit tired. But he enjoyed it… I'd say to him, ‘Did you enjoy it?' ‘Oh yeah, it was good”' (participant 3)*.

The garden provided cognitive stimulation to patients in several ways. For some participants this was seen through the need for patients to manage the cognitive demands of the different environment.

“*Yeah well, like I said, you need stimulation, so not only does it stimulate you physically but also mentally. Having to deal with different environments, and that's a good example of that because of the changes in the texture of the ground as you're walking along with the bark, the gravel, and the concrete” (participant 4)*.

“*Just the general layout of it, learning the plants and the bird boxes” (patient)*.

Other participants reflected on the opportunities provided by the garden features to reminisce, recall and explore.

“*I think it must bring some form of memory back, especially if they've been in the garden in their lifetime. I know when I've picked mint out there and shown Bill and he does smell it. It's the sensory behind it that's good for them” (participant 6)*.

“*I think she just liked exploring” (participant 5)*.

“*And I'll go and I'll just squeeze it and I'll say, what does that smell like? And he will say something” (participant 2)*.

#### 3.4.4 Barriers and facilitators

Family members discussed a range of barriers and facilitators to the garden's use. The requirement for staff to support patient access was identified by most carers, with some seeing it as a positive that allowed patients safe experiences in the garden that they would otherwise not be able to have.

“*If he didn't have anyone's hands to hold and he couldn't use his frame then he'd struggle, he wouldn't be able to do it” (participant 3)*.

However, other participants felt that getting access to the locked garden was a barrier limiting patient's use.

“*It wasn't used much cause it was locked. People couldn't just wander out there when they felt like it” (participant 5)*.

The challenge of balancing features of the garden for different needs was discussed by several participants. Consideration around how to challenge the mobility of more able patients while still making the garden safe for less mobile patients was discussed.

“*Yes. Perhaps you want more of one level there where people who are strong can do a bit more walking up slopes and that sort of thing… And people who aren't that can just walk around, but bypass that sort of thing” (participant 5)*.

Almost all family members had good suggestions about additional features to support patients to engage with occupations in the garden including attracting birds, types of plants, familiar garden entertainment features like barbeques, places to explore and puzzles and games.

The variety of features was also something participants considered valuable in making the garden engaging.

“*I've mentioned it to my mum... I said to her, ‘They got this huge big garden that's concrete and grass and plants. And they walk around the seats and that. But down the other side, there's a beautiful little smaller garden with steps, a little bridge'… ‘They've got all the herbs and that down there.' And I said, ‘Not that they eat them or anything or use them for cooking.' But you can feel them. And those rabbits ears, they're lovely and soft and fluffy… Mum was amazed” (participant 6)*.

Half of the participants felt that the garden would be better if it had been larger, however, one participant felt the small size provided a sense of security.

## 4 Discussion

This evaluation revealed that the redevelopment of the garden space for people with dementia on an acute unit was regarded positively by families and most staff agreed there were benefits for patients. Furthermore, our interrupted time series analysis showed that redevelopment of the garden space did not lead to increased falls or challenging behaviour incidents on the unit. The initial intention of the garden was to (1) enable patients to spend time in a therapeutic outdoor environment, and (2) provide opportunities for mobility training, maintenance and rehabilitation. We did not collect quantitative data to determine whether these aims were met in this study and our focus was on more clinical outcomes and patient and family experience. Our interview data revealed that there were a variety of benefits experienced (such as reinforcing self-identity and cognitive stimulation) which we did not initially set out to achieve.

Staff concerns regarding risk of harm were identified in this study and are commonly recognised as a barrier to garden access (Barrett et al., 2019; Evans et al., 2019; van den Berg et al., 2020; Fielder and Marsh, 2021). Perceived risk of falls is frequently identified as a concern despite little evidence that gardens do indeed increase the risk of falls for older people (van den Berg et al., 2020). Our study identified a drop in falls at the time the garden was introduced. However, our findings should be interpreted with caution due to the small numbers of patients and falls incidents. Furthermore, use of the garden was limited; we estimate that the garden was used ~4 times per day by the 5–10 patients who could be present on the unit on any given day. Nevertheless, it is encouraging that the redevelopment of the garden space did not increase falls occurring on the unit given staff concerns identified. In addition, no falls occurred in the garden itself in the 6 months following it's opening. Some research suggests that outdoor spaces may have a positive impact on overall falls rates (Lai et al., 2023) and that there can be reductions in falls risk contributors such as agitation and antipsychotic medication use (Detweiler et al., 2009). More investigation into the actual risk of falls in outdoor spaces may be appropriate to identify if persisting staff concerns over falls in gardens is warranted.

Our study identified that families and staff have positive beliefs regarding the benefits of the garden. However, our study is likely to be underpowered to detect differences in quantitative outcomes. This reflects other studies identifying positive qualitative findings but limited quantitative outcomes for the use of outdoor spaces (Murroni et al., 2021). Falls, challenging behaviours and other issues experienced by people with dementia are often complex and multifactorial (Eriksson et al., 2007; Van Mierlo et al., 2010). Preferences for accommodating people with dementia in small home like settings makes it difficult to conduct high quality quantitative studies with large numbers of participants (Edwards et al., 2013). This makes interventions such as outdoor spaces which may impact on the lives of people with dementia through many different factors difficult to study. New technologies, such as the use of wearable devices, are being used to investigate a range of outcomes for people with dementia (Cote et al., 2021). Use of new technologies may assist researchers to complete higher quality studies in areas such as the use of outdoor spaces that are difficult to investigate.

The importance of staff supporting the access and engagement of people with dementia to use outdoor spaces continues to be an important component of research in this area. We found that allied health professionals supported patients to use the garden space. This is contrary to residential care home settings where few studies report allied health involvement and use (Ng et al., 2023). This outcome may reflect the respective levels of allied health staffing availability in mental health units when compared to residential care home settings. Lack of time was seen as the biggest barrier to staff supporting patients to access the garden. Previous studies have made recommendations that usual indoor care tasks take place outdoors (van der Velde-van Buuringen et al., 2021) which could help to mitigate demands on time. In the current study, some staff preferred the garden as they believed it provided a more stimulating environment or an alternative space when distressing events were occurring inside the unit. In addition, most staff believed that the key benefits were exposure to sensory experiences, activity and being in nature. This indicates that for staff there may be a delineation between daily tasks being part of indoor spaces and outdoor spaces being places for pleasure.

It has been recommended that staff receive training and education around the use of outdoor spaces for people with dementia (Gonzalez and Kirkevold, 2015; van den Berg et al., 2020), Models such as the Garden-Use Model (Grant and Wineman, 2007) also identify the important role that the attitudes of the organisation and staff play in the use of garden spaces (Grant and Wineman, 2007). In our study, the majority of survey participants had worked on the unit for < 3 years and had not used the garden space themselves. Use of co-design strategies may support the development of policies and procedures supporting garden use that are more acceptable and usable by end users. However, the effectiveness of co-design strategies to implement long term change is yet to be demonstrated (Slattery et al., 2020).

### 4.1 Limitations

The findings from this study should be considered exploratory and interpreted with caution for two main reasons. Models which categorise people with behavioural and psychological symptoms of dementia into tiers describe those people at tiers six and seven as having the most serious of symptoms (Brodaty et al., 2003). People in tier six and seven are estimated to form < 1% of the population of people with dementia (Brodaty et al., 2003). This study took place on a unit caring for this population and as such the findings may not be generalisable to a broader patient population with dementia who have milder symptoms. However, a strength of the study is that there is limited research conducted related to garden use and people considered to have tier six or seven characteristics and the findings are likely to be of high interest to similar settings. Also, the small numbers of patients on this unit mean that the study is very vulnerable to the changing characteristics of the population as different patients are admitted and discharged over time. There were no adjustments made for changes in the characteristics of the population.

This study utilised measurements taken over a period of 12 months and therefore it is possible that seasonal effects may impact the outcomes. For example, it is possible that people might be less active over winter due to rain and cold temperatures. However, data from Australia and the United States on falls in hospitals suggest a tendency to be relatively consistent across seasons (Stevens et al., 2007; Welfare, 2023). It has also been suggested that high temperatures may cause distress and increase the rates of behavioural and psychological symptoms of dementia (Cornali et al., 2004). However, this is less likely to be applicable to the temperature-controlled hospital environment. Furthermore, acute hospitals are dynamic spaces and changes to care, documentation and policies occur regularly. This is a threat to the validity of interrupted time series studies where there is no prospective control group. Finally, it is possible that increased use of the garden may have occurred when the garden opened due to the novelty of having a new space, but that this usage decreased once it was no longer new. However, there are no observational data available on the frequency of use of the garden.

## 5 Conclusion

This study shows that garden and outdoor spaces are generally highly regarded by families and staff, but there are multiple barriers to use. Although staff may be concerned about risks stemming from use of the garden environment, falls did not increase on the unit and no falls occurred in the garden area in the 6 months of the study. Given the range of benefits that come with access to outdoor environments for people living with dementia, further research is justified to understand staff concerns around risks and how to address this and other barriers to garden use. New technologies may be beneficial in assisting researchers to investigate outcomes.

## Data availability statement

The datasets presented in this article are not readily available because, consent was given for data to be used for this study only. Requests to access the datasets should be directed to CALHN Research Services, Health.CALHNResearchGovernance@sa.gov.au.

## Ethics statement

The studies involving humans were approved by Central Adelaide Local Health Network Human Research Ethic Committee (CALHN HREC). The studies were conducted in accordance with the local legislation and institutional requirements. The Ethics Committee/Institutional Review Board waived the requirement of written informed consent for participation from the patient participants or the participants' legal guardians/next of kin because de-identified, routinely collected data was utilised. Written informed consent for participation was gained from interview participants. The front page of the survey provided information about the study and stated “Completion of the questionnaire will be taken as indicating your consent to participate”. Survey participant who consented completed the survey, those who did not consent did not complete the survey.

## Author contributions

LN: Conceptualisation, Data curation, Formal analysis, Investigation, Methodology, Project administration, Software, Validation, Writing—original draught, Writing—review & editing, Resources. SDy: Methodology, Supervision, Writing—original draught, Writing—review & editing. SDa: Writing—original draught, Writing—review & editing, Supervision. KL: Writing—original draught, Writing—review & editing, Supervision, Conceptualisation, Formal analysis, Investigation, Methodology, Resources, Software, Validation.
