# Supplementary material for: Impact of an improved outdoor space on people with dementia in a hospital unit
Source: Front Dement. 2024 Jun 6;3:1404662. doi: 10.3389/frdem.2024.1404662 (PMC11285644; doi:10.3389/frdem.2024.1404662)
Supplement: Supplementary file 1 [file Data_Sheet_1.docx]

STAFF SURVEY QUESTIONS

My job role is:

- Nursing
- Psychiatric/Medical
- Allied Health
- Other___________

(or split further if useful)

I have worked on the ward for:

- Less than 12 months
- 1-2 years
- 2-3 years
- 3-4 years
- 4-5 years
- 5-10 years
- Over 10 years

I have spent time in the space with a consumer:

- Yes/No

I have seen someone else spend time in the space with a consumer:

- Yes/No

How often do you see someone in the garden:

- Every day
- Most days
- Once or twice a week
- Rarely
- Never

I have seen the following types of people in the garden (tick all that apply)

- Consumers
- Nurses
- Doctors
- Allied Health
- Consumer’s families
- Consumer’s friends
- Other__________

What types of activities have you done with consumers or seen them do in the Happy Trails Garden? (tick all that apply)

- Animal activities (eg. watching birds)
- Clinical activities (eg. participating in an assessment, therapy)
- Food activities (eg. eating a snack, having a drink)
- Physical exercise or games (eg. walking, quoits)
- Gardening activities (eg. weeding, watering, sweeping, fertilising, pick flowers)
- Passive activities (eg. relaxing, sunbathing, sitting)
- Social activities (eg. chatting, reminiscing, group activities)
- Other activities (eg. exploring, playing a game, singing, talking on the phone)

What specific activities have you done with consumers or seen them do in the Happy Trails Garden? (tick all that apply)

- Talking to others
- Participating in groups
- Playing games
- Looking after plants
- Practicing walking
- Practicing steps
- Looking at garden/scenery
- Smelling plants
- Tasting plants
- Sweeping/tidying the garden
- Reminiscing
- Sunbathing
- Picking plants/flowers
- Watering
- Exercising
- Completing assessments
- Other________________

What factors influence your ability to use the garden? (tick all that apply)

- Availability of time
- Weather
- Amount of people already in the garden
- Concerns about own safety
- Concern about consumer safety
- Confidence in offering therapeutic activities in the garden
- Own preference to spend time outdoors

What sort of behaviours would prompt you to consider taking a consumer into the garden? (tick all that apply)

- Concerns about consumer deconditioning
- Consumer asks to go out
- Consumer is restless
- Consumer is sad
- Consumer is angry
- Consumer is bored
- Consumer is happy
- Consumer is looking at garden
- Consumer is pacing
- To develop rapport
- Other__________

I think the Happy Trails Garden has positive impacts on consumers mood and/or behaviour while they are using it:

- Strongly agree
- Agree
- Neutral
- Disagree
- Strongly disagree

I think the Happy Trails Garden has positive impacts on consumers mood and/or behaviour after they have used it:

- Strongly agree
- Agree
- Neutral
- Disagree
- Strongly disagree

I think most consumers like the garden:

- Strongly agree
- Agree
- Neutral
- Disagree
- Strongly disagree

I think most families and visitors like the garden:

- Strongly agree
- Agree
- Neutral
- Disagree
- Strongly disagree

I think most staff on our ward have positive opinion about using the garden:

- Strongly agree
- Agree
- Neutral
- Disagree
- Strongly disagree

If someone from another ward or facility asked you, you would recommend that they have a garden like the Happy Trails Garden:

- Strongly agree
- Agree
- Neutral
- Disagree
- Strongly disagree

Safety concerns I have about consumers spending time in the garden are: (tick all that apply)

- Aggression
- Challenging behaviour
- Cold
- Cuts or abrasions
- Falls
- Heat stress
- Infection
- Injuries
- Sunburn
- Other__________________

I think that consumers spending time in the garden is:

- Very safe
- Safe
- Medium
- Risky
- Very risky

In what situation might you choose spending time in ‘Happy Trails’ garden over the circular courtyard?

_______________________________

In what situation might you choose spending time in the circular courtyard over the ‘Happy Trails’ garden?

_______________________________

Do you think the addition of the Happy Trails garden has contributed to a more positive work environment? If so, in what ways?

__________________________________

In what ways does the Happy Trails Garden improve the ward space (tick all that apply)

- Activity options
- Assessment options for staff
- Attractive view
- Attracts birds and insects
- Cognitive support
- Connect with nature
- Emotional support
- Fresh air
- Intervention options for staff
- Sensory experiences
- Sunlight
- Other______________

Are there any suggestions you would make for the Happy Trails Garden?

___________________________________
